# Clinical value of ACR O-RADS combined with CA125 in the risk stratification of adnexal masses

**DOI:** 10.3389/fonc.2024.1369900

**Published:** 2024-08-30

**Authors:** Rui-Ke Pan, Shu-Qin Zhang, Xian-Ya Zhang, Tong Xu, Xin-Wu Cui, Ran Li, Ming Yu, Bo Zhang

**Affiliations:** ^1^ Department of Medical Ultrasound, Shanghai East Hospital, Nanjing Medical University, Shanghai, China; ^2^ Department of Medical Ultrasound, The First People’s Hospital of Lianyungang, Lianyungang, Jiangsu, China; ^3^ Department of Medical Ultrasound, Tongji Hospital, Tongji Medical College, Huazhong University of Science and Technology, Wuhan, China; ^4^ Department of Medical Ultrasound, The First Affiliated Hospital of Xinxiang Medical University, Xinxiang, China

**Keywords:** O-RADS, CA125, adnexal masses, ultrasound, malignancy risk

## Abstract

**Purpose:**

To develop a combined diagnostic model integrating the subclassification of the 2022 version of the American College of Radiology (ACR) Ovarian-Adnexal Reporting and Data System (O-RADS) with carbohydrate antigen 125 (CA125) and to validate whether the combined model can offer superior diagnostic efficacy than O-RADS alone in assessing adnexal malignancy risk.

**Methods:**

A retrospective analysis was performed on 593 patients with adnexal masses (AMs), and the pathological and clinical data were included. According to the large differences in malignancy risk indices for different image features in O-RADS category 4, the lesions were categorized into groups A and B. A new diagnostic criterion was developed. Lesions identified as category 1, 2, 3, or 4A with a CA125 level below 35 U/ml were classified as benign. Lesions identified as category 4A with a CA125 level more than or equal to 35 U/ml and lesions with a category of 4B and 5 were classified as malignant. The sensitivity, specificity, positive predictive value (PPV), negative predictive value (NPV), accuracy, and area under the curve (AUC) of O-RADS (v2022), CA125, and the combined model in the diagnosis of AMs were calculated and compared.

**Results:**

The sensitivity, specificity, PPV, NPV, accuracy, and AUCs of the combined model were 92.4%, 96.5%, 80.2%, 98.8%, 94.1%, and 0.945, respectively. The specificity, PPV, accuracy, and AUC of the combined model were significantly higher than those of O-RADS alone (all *P* < 0.01). In addition, both models had acceptable sensitivity and NPV, but there were no significant differences among them (*P* > 0.05).

**Conclusion:**

The combined model integrating O-RADS subclassification with CA125 could improve the specificity and PPV in diagnosing malignant AMs. It could be a valuable tool in the clinical application of risk stratification of AMs.

## Introduction

Adnexal masses (AMs) are commonly found in women, with a reported incidence ranging from 6% to 17% (1, 2). Accurate assessment of AMs as benign or malignant is crucial in individual management. AMs suspected to be malignant should be referred immediately to a specialized oncology center for appropriate treatment (3–5). Conversely, AMs that are considered benign require a different management: if the patient has obvious or intolerable clinical symptoms related to the mass, surgical excision may be beneficial (6). However, if the patient has no obvious clinical symptoms, surgical treatment may be costly and carry the risk of complications. In such cases, conservative treatment may be a preferable option (7).

Ultrasound (US) is the primary imaging modality for the preoperative assessment of the malignancy of AMs, providing essential information for the clinical management of patients. It is widely believed that the subjective assessment by US experts is the most accurate (3), but the number of experts is limited. Therefore, a series of ultrasound-based diagnostic models have been developed to aid in diagnosing malignant AMs and have been externally validated (8–12). However, variability among ultrasound reports is also an issue, as it somewhat limits the effectiveness of ultrasound assessment and impacts patients’ clinical management (13). In 2020, the American College of Radiology (ACR) published the Ovarian-Adnexal Reporting and Data System (O-RADS) (12), which introduced standardized lexicons to unify descriptors and reduce ambiguity in US reports. The O-RADS categorizes AMs into six categories, ranging from 0 to 5, covering all risk levels from normal to highly malignant, and offers corresponding management strategies to standardize clinical management. O-RADS is more sensitive than other risk stratification systems, but its specificity is rather not outstanding (14–17). The ACR released an updated version of O-RADS, adding new descriptors to the original, including “bilocular” for cystic lesions, “acoustic shadowing” for smooth solid lesions, and additional descriptors for classic benign lesions, aiming to further improve the diagnostic specificity for low-risk lesions (18). However, effective clinical validation for the O-RADS (v2022) is still lacking. Furthermore, the malignancy rate for O-RADS 4 lesions ranges from 10% to 50%, a broad range that hinders the precise clinical management of these lesions. For O-RADS 4 lesions, the guidelines suggest that MRI can be chosen for further evaluation. However, there are still some lesions that can be misclassified due to a misunderstanding of the dictionary definition of solid tissue (19). Cao et al. (20) have explored the subclassification of O-RADS 4 category lesions, considering that this approach could improve both specificity and accuracy. However, no studies have yet demonstrated whether the combination of subclassification and O-RADS (v2022) can further enhance diagnostic performance and optimize risk stratification.

Tumor biomarkers are pivotal in detecting ovarian cancer, complementing the limitations of conventional imaging approaches and providing adequate clinical diagnostic information. Carbohydrate antigen 125 (CA125) has emerged as the most promising marker for screening and monitoring ovarian cancer (21). Although elevated levels of CA125 are also detected in physiologic and benign conditions such as endometriosis (22), inflammations, and pregnancy (23), which decreases its specificity, CA125 remains superior to most novel biomarkers in postmenopausal women, including human epididymis protein 4 (HE4) (24). HE4 is considered the most valuable tumor biomarker for ovarian cancer, second only to CA125, offering good specificity (25). However, HE4 is rarely expressed in mucinous epithelial and germinal cancers, resulting in insufficient diagnostic sensitivity (26). Moreover, HE4 levels may be raised by smoking and reduced by taking oral contraception. Therefore, HE4 values in these individuals should be interpreted with caution (27, 28). Studies have highlighted that the combined use of CA125 and HE4 exhibits certain value in diagnosing ovarian cancer, which led to the creation of the ROMA algorithm. ROMA integrates CA125, HE4, and the patient’s menopausal status to provide a more accurate prediction of ovarian tumors. However, studies are divided on the diagnostic efficacy of ROMA (29, 30). Some scholars (31) question its superiority, especially when compared to the standalone use of CA125, as ROMA and HE4 have not shown significant advantages. Further analysis shows that in postmenopausal women, CA125’s diagnostic efficacy seems to surpass that of HE4. This has led the author to conclude that HE4 and ROMA might not significantly enhance the diagnosis of ovarian cancer. Furthermore, some literature reports that combining the O-RADS system with tumor markers (like CA125) may enhance diagnostic accuracy (32, 33). However, it is important to note that these studies primarily focus on combining all O-RADS categories with CA125, while the O-RADS 4 lesions, which are most prone to false-positive diagnoses, receive inadequate attention. Therefore, it is necessary to explore how to optimize combined diagnostic strategies to enhance the diagnostic efficacy for ovarian cancer, especially for O-RADS categories with wide risk ranges.

Thus, this study aimed to develop a combined diagnostic model that integrated the subclassification of O-RADS (v2022) with CA125 and to ascertain whether the combined approach can offer superior diagnostic efficacy compared to using O-RADS (v2022) alone in assessing adnexal malignancy risk.

## Materials and methods

### Patients

The retrospective single-center study was approved by the First People’s Hospital of Lianyungang Ethics Committee. Informed consent was waived. From February 2020 to October 2021, patients with AMs who received surgery and had determined pathological results were collected. The inclusion criteria were as follows: i) patients diagnosed with AM on US, ii) patients who underwent CA125 examination before surgery, and iii) patients with no prior history of ovariectomy or chemotherapy. The exclusion criteria were as follows: i) an interval greater than 30 days between US and surgery, ii) patients with uncertain pathological results, iii) patients who are pregnant, and iv) patients with ascites due to other diseases.

### US examination

All the enrolled patients underwent transvaginal US by experienced radiologists. If the mass was too large to be entirely evaluated, transabdominal US was additionally performed. The US equipment included LOGIQ E9 (GE Healthcare, Milwaukee, WI, USA) and Voluson E10 (GE Healthcare, Milwaukee, WI, USA). An RIC5-9-D probe (GE Healthcare, Milwaukee, WI, USA) and a C1-6-D probe were used.

### Retrospective images analysis

Clinical and pathological information was collected from electronic medical records. All US images were independently reviewed by two radiologists with at least 5 years of experience in gynecological US who were blinded to the pathologic results. If there was a disagreement between the two radiologists, all images were discussed in detail until a consensus was reached. If a patient had more than one AM, the one with the most complex US morphology was enrolled. According to the descriptor terms of the O-RADS (v2022) (18, 34), the following characteristics were acquired for each AM: maximum diameters of the lesion, size of the solid component, external contour, number of locules, internal margin or walls, acoustic shadowing, number and size of papillary projections (pps), vascularity, ascites, and peritoneal nodules. Previous studies (20, 35) have shown that diagnostic accuracy improves when considering O-RADS 4 to 5 as indicative of malignancy. Therefore, in this study, masses categorized as O-RADS 1 to 3 were designated as benign, while those classified as O-RADS 4 to 5 were classified as malignant. Borderline tumors were considered malignant. The level of serum CA125 was measured within 14 days before surgery. CA125 ≥35 U/ml was considered positive (21).

### Diagnostic criteria of O-RADS (v2022) subclassification combined with CA125

In this study, for the combined model, O-RADS 4 was firstly subclassified into two groups: categories 4A and 4B. Bi- or multilocular cysts without solid components (any color score) and unilocular cysts <4 pps (any color score) or with solid components (any color score) were defined as category 4A. Bi- or multilocular cysts with solid components (color score 1–2) and smooth solid lesions (color score 2–3) were defined as category 4B. For the combined model, O-RADS categories 1, 2, 3, and 4A with CA125 <35 U/ml were defined as benign masses. O-RADS category 4A with CA125 ≥35 U/ml, O-RADS 4B, and O-RADS 5 were defined as malignant masses.

### Statistical analysis

The sample size of this study was 593 cases. SPSS (version 26.0; IBM, Armonk, NY, USA) and MedCalc (version 19.0; MedCalc software) software were used for the statistical analyses. Continuous variables were expressed as mean ± standard deviation and compared by independent samples *t*-test. Categorical variables were expressed as frequencies and percentages, and comparisons between the two groups were made using the chi-square test. Accuracy, specificity, sensitivity, positive predictive value (PPV), and negative predictive value (NPV) were calculated to compare the diagnostic performance of the combined model with O-RADS (v2022) or CA125 alone in differentiating benign and malignant AMs. The McNemar’s test was used to compare the differences between the two methods. The area under the curve (AUC) was compared by the Delong method. *P <*0.05 was considered significant.

## Results

### Participant and lesion characteristics

**Figure 1:**
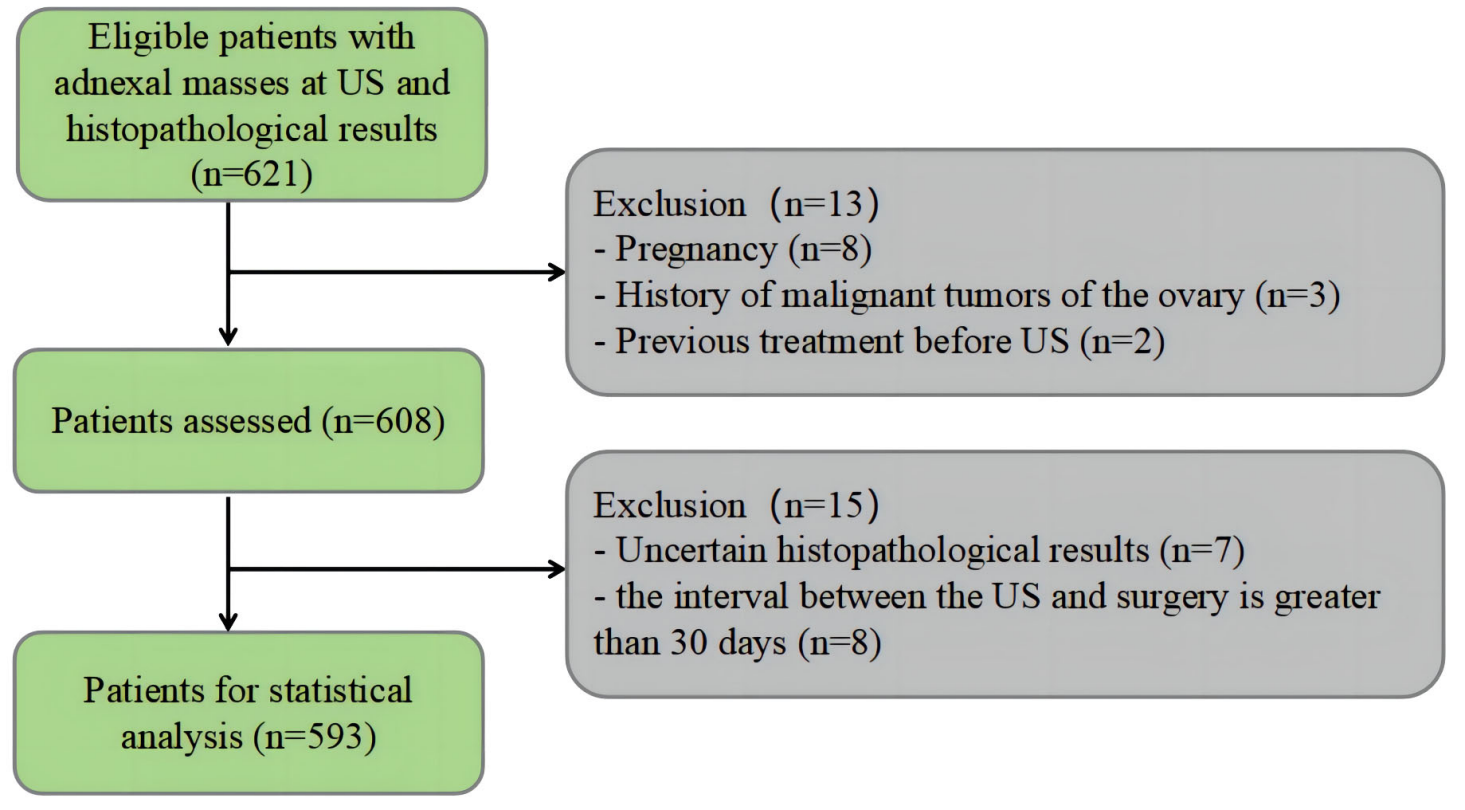
Flowchart shows the patient selection with adnexal mass. US, ultrasound.

A total of 593 lesions in 593 patients were included in this study. There were 514 (86.7%) benign lesions and 79 (13.3%) malignant lesions. The flowchart of patient selection is shown in **Figure 1**. The clinical baseline characteristics and CA125 levels are shown in **Table 1**. In comparison to benign tumors, malignant tumors were more commonly found in older postmenopausal women (*P* < 0.01).

**Table 1:** General characteristics of the patients with adnexal masses (*n* = 593).

Characteristics		Final diagnosis		*P-*value
		Benign	Malignant	
		(*n* = 514)	(n = 79)	
Age, years (mean ± SD)		37.2 ± 12.2	46.4 ± 15.8	<0.001
Postmenopausal	Yes	30	43	<0.001
	No	484	36	
CA125	Increased	159	59	
	Normal	355	20	

CA125, carbohydrate antigen 125.

### Results of the O-RADS (v2022) classification

In the evaluated 593 masses, 417 (70.3%) were categorized as O-RADS 2, 56 (9.4%) were categorized as O-RADS 3, 82 (13.8%) were categorized as O-RADS 4 (43 were 4A and 39 were 4B), and 38 (6.4%) were categorized as O-RADS 5. The O-RADS categories and histologic diagnosis are summarized in **Table 2**. There were five malignancies classified as O-RADS 2 or O-RADS 3 lesions. Three false-negative cases were unilocular cysts including two cysts classified as O-RADS 2 and one cyst with a diameter >10 cm classified as O-RADS 3. Histology showed that one case was a cystic adult granulosa cell tumor and two cases were borderline serous cystadenoma. Two false-negative cases were bilocular cysts that showed homogeneous hypoechoes with a diameter >10 cm and were diagnosed as typical ovarian endometrioma which was classified as O-RADS 3. Histology showed that these two cases were borderline serous cystadenoma.

**Table 2:** The O-RADS(v2022) classification according to specific histologic diagnosis of 593 adnexal masses.

Histologic diagnosis	O-RADS (v2022) classification					Total
	2	3	4A	4B	5	
Benign adnexal masses	415	53	34	11	1	514
Follicular cyst	6	1	0	0	0	7
Corpus luteum	23	2	1	0	0	25
Simple cyst	24	4	2	0	0	30
Endometrioma	167	8	2	0	1	178
Mature teratoma	82	5	6	3	0	96
Hydrosalpinx	20	0	1	1	0	22
Mesosalpinx cyst	34	1	0	0	0	35
Pelvic inflammatory disease	27	2	2	0	0	31
Serous cystadenoma	16	10	6	3	0	35
Mucinous cystadenoma	12	13	11	0	0	36
Ovarian theca-fibroma	3	6	1	1	0	11
Other benign adnexal masses	1	1	3	3	0	8
Malignant adnexal masses	2	3	9	28	37	79
Serous cystadenocarcinoma	0	0	2	8	19	29
Mucinous cystadenocarcinoma	0	0	2	0	0	2
Borderline tumor	1	3	2	9	3	18
Germ cell tumor	0	0	0	2	1	3
Sex cord-stromal tumor	1	0	1	0	2	4
Endometrioid carcinoma	0	0	0	0	3	3
Clear cell tumor	0	0	2	4	0	6
Metastatic cancer	0	0	0	3	7	10
Other malignant adnexal masses	0	0	0	2	2	4
Total	417	56	43	39	38	593

O-RADS, Ovarian-Adnexal Reporting and Data System; v, version.

### Classification of the O-RADS (v2022) subclassification combined with CA125

The subcategories of O-RADS 4 lesions included 1) bilocular cyst without a solid component, irregular, any size, and any color score; 2) multilocular cyst without a solid component (a. smooth, ≥10 cm, color score <4; b. smooth, any size, color score 4; c. irregular, any size, any color score); 3) unilocular cyst with a solid component, <4 pps or a solid component not considered a pp, any size; 4) bi- or multilocular cyst with a solid component, any size, color score 1–2; and 5) solid lesion, non-shadowing, smooth, any size, color score 2–3, presenting different malignancy rates, which were 16.7% (1 out of 6 lesions), 22.7% (5 out of 22 lesions), 20% (3 out of 15 lesions), 66.7% (20 out of 30 lesions), and 88.9% (8 out of 9 lesions), respectively. After the subclassification, it was evident that the malignancy rate for category 4B was significantly higher at 71.8%, compared to 20.9% for category 4A (**Table 3**). This substantial difference indicated more effective risk stratification (*P* < 0.01).

**Table 3:** Image characteristics of the 593 lesions.

		B	M	Total	Malignant rate (100%)
O-RADS (v2022) categories and lexicon descriptors					
Categories	Lexicon descriptors				
2		415	2	417	0.48
	Simple cyst (<10 cm)	34	2	36	
	Unilocular, smooth, non-simple cyst/bilocular, smooth cyst (<10 cm)	80	0	80	
	Typical benign ovarian lesion (<10 cm)	278	0	278	
	Typical benign extraovarian lesion (any size)	23	0	23	
3		53	3	56	5.36
	Typical benign ovarian lesion (≥10 cm)	8	2	11	
	Uni- or bilocular, smooth (≥10 cm)	5	1	5	
	Unilocular, irregular (any size)	5	0	5	
	Multilocular, smooth, <10 cm, CS <4	22	0	22	
	Solid lesion, ± shadowing, Smooth, any size, CS 1	7	0	7	
	Solid lesion, shadowing, smooth, any size, CS 2–3	6	0	6	
4		45	37	82	45.12
4A	Bilocular cyst without a solid component, irregular, any size, any CS	5	1	6	20.9
	Multilocular cyst without a solid component	17	5	22	
	Unilocular cyst with a solid component, <4 pps or solid component not considered a pp, any size	12	3	15	
4B	Bi- or multilocular cyst with a solid component, any size, CS 1–2	10	20	30	71.8
	Solid lesion, non-shadowing, smooth, any size, CS 2–3	1	8	9	
5		1	37	38	97.37
	Unilocular cyst ≥4 pps, any size, any CS	0	2	2	
	Bi- or multilocular cyst with a solid component, any size, CS 3–4	1	1	2	
	Solid lesion, ± shadowing, smooth, any size, CS 4	0	10	10	
	Solid lesion, irregular, any size, any CS	0	16	16	
	Ascites and/or peritoneal nodules	0	8	8	

O-RADS, Ovarian-Adnexal Reporting and Data System; v, version; B, benign; M, malignant; CS, color score; pps, papillary projections.

After the adjustment using O-RADS combined with CA125, within the category of O-RADS 4 lesions, 28 lesions were accurately classified as benign and 1 malignant lesion was incorrectly classified as benign (**Table 4**).

**Table 4:** Comparison of the assessment results of O-RADS 4 lesions between O-RADS (v2022) alone and O-RADS (v2022) combined with the CA125 model.

Group	Benign	Malignant	Total	Malignant rate (100%)
O-RADS (v2022) 4	45	37	82	
4A	34	9	43	20.9
4B	11	28	39	71.8
O-RADS (v2022) subclassification + CA125				
O-RADS 4	45	37	82	
4A + CA125 <35 U/ml	28	1	29	3.45
4A + CA125 ≥35 U/ml, 4B	17	36	53	67.9

O-RADS, Ovarian-Adnexal Reporting and Data System; v, version; CA125, carbohydrate antigen 125.

### Diagnostic performance of the CA125, O-RADS (v2022), and O-RADS (v2022) subclassification combined with CA125

**Figure 2:**
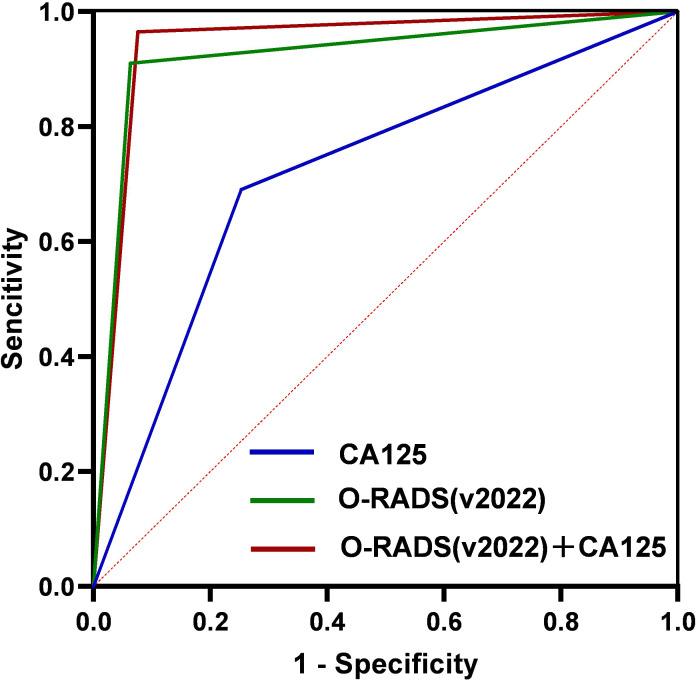
ROC curve for CA125, O-RADS (v2022), and O-RADS (v2022) combined with CA125. ROC, receiver operating characteristic; CA125, carbohydrate antigen 125; O-RADS, Ovarian-Adnexal Reporting and Data System; v, version.

The AUC values of CA125 alone, O-RADS (v2022) alone, and O-RADS (v2022) subclassification combined with CA125 were 0.719, 0.924, and 0.945, respectively (**Figure 2**). The sensitivity, specificity, accuracy, PPV, and NPV of the three are shown in **Table 5**. The specificity, PPV, accuracy, and AUC of the O-RADS subclassification combined with CA125 were considerably higher than those of O-RADS alone (*P* < 0.01). In addition, both models had good sensitivity and NPV, but there were no remarkable differences between them (*P* > 0.05).

**Table 5:** Diagnostic efficacy of CA125, O-RADS (v2022), and O-RADS (v2022) combined with CA125.

Assessment method	SE	SP	PPV	NPV	AC	AUC	*P*
CA125	0.747	0.691	0.271	0.946	0.698	0.719	<0.001^a^
O-RADS (v2022)	0.937	0.911	0.617	0.989	0.914	0.924	0.009^b^
O-RADS (v2022) + CA125	0.924	0.965	0.802	0.988	0.941	0.945	<0.001^c^

O-RADS, Ovarian-Adnexal Reporting and Data System; v, version; CA125, carbohydrate antigen 125; SE, sensitivity; SP, specificity; PPV, positive predictive value; NPV, negative predictive value; AC, accuracy, P^a^, CA125 compared with O-RADS (v2022); P^b^, O-RADS (v2022) compared with O-RADS (v2022) combined with CA125; P^c^, CA125 compared with O-RADS (v2022) combined with CA125.

## Discussion

The ACR O-RADS offers a precision risk stratification system, and its diagnostic power for AMs has been confirmed in various studies (16, 35, 36). However, despite the high sensitivity, its specificity for identifying benign and malignant lesions is moderate (20, 37). This moderate specificity could lead to overtreatment in clinical settings (14, 17, 35). Therefore, in this study, we developed and evaluated a diagnostic model that integrated O-RADS and CA125 for AM classification. It revealed that O-RADS combined with the CA125 model exhibited superior diagnostic performance compared to O-RADS alone, with an AUC of 0.945 versus 0.924 (*P* = 0.009). Moreover, it improved the diagnostic specificity and PPV and retained a high sensitivity and NPV. To the best of our knowledge, our study is the first to subclassify O-RADS 4 lesions using the updated O-RADS (v2022) and to integrate this with CA125 levels to assess its efficacy in differentiating between benign and malignant AMs.

The O-RADS (v2022) has been demonstrated to improve the diagnostic specificity for AMs. A study by Su et al. showed that O-RADS (v2022) had higher accuracy (89.4% vs. 84.4%) and specificity (86.1% vs. 79.5%) than O-RADS version 1 (v1). In our study, the specificity of O-RADS (v2022) was 91.1%, which was slightly higher than the 86.1% in Na Su’s study (38). The difference may be due to the varying proportions of cystadenomas in the two studies (26% vs. 14%). Cystadenomas were often characterized by either unilocular cysts with solid elements or multilocular cysts lacking solid components (large size, high color score, or irregular surface) on US. These lesions are typically categorized as O-RADS 4. When > O-RADS 3 is used as a predictor of malignancy, the lesions are often classified in the malignant category, which reduces diagnostic specificity. The relatively low prevalence of cystadenomas in this study might explain their higher specificity.

In this study, 94.6% (53/56) of the benign tumors in O-RADS 3 and 97.3% (37/38) of the malignant tumors in O-RADS 5 exhibited excellent specificity. In addition, 28 benign lesions in O-RADS 4 were accurately identified using the combined diagnostic model, significantly improving the diagnostic specificity. These 28 lesions included 6 serous cystadenomas (unilocular solid masses, color score 1–2) and 11 mucinous cystadenomas (multilocular cysts without solid components, 8 cases with a maximum diameter >10 cm, 3 cases with irregular inner walls). According to the O-RADS classification criteria, these lesions are mostly categorized as O-RADS 4. Patients with these lesions might be advised to undergo an MRI or be referred to an ultrasound specialist for further evaluation and be referred to a gynecologic oncologist for management. However, our combined diagnostic approach correctly classified these nodules as benign, saving patients both time and cost by avoiding the need for MRI or reassessment by ultrasound specialists. Additionally, it aids clinicians in making clinical decisions regarding follow-up timing (if surgery is not chosen), selection of surgical strategies (considering open surgery if malignancy is suspected), and the waiting time for surgery.

Although most US features of O-RADS 4A typically suggest benign conditions, exclusively relying on US may result in overlooking malignant cases. The integration of CA125 into the diagnostic process can potentially mitigate the risk of false negatives arising from subclassification. A previous study showed that combining US with biomarkers significantly enhanced the accuracy of predicting ovarian cancer (39). In our study, six cases initially subclassified as O-RADS 4A were accurately identified as malignant owing to elevated CA125 levels. These included five cases of multicystic lesions without solid components (comprising three mucinous cystadenocarcinomas and two high-grade serous carcinomas) and one case of a unilocular cyst with a solid component identified as clear cell carcinoma. A previous study showed that CA125 is elevated in approximately 57.6% of clear cell carcinoma cases (40). Although CA125 levels are usually not high in primary ovarian mucinous carcinoma, it is important to note that approximately 80% of ovarian mucinous carcinomas are actually metastatic. In cases of metastatic ovarian cancer, CA125 levels tend to be significantly elevated (41). For metastatic ovarian mucinous carcinoma, the primary site is most commonly the gastrointestinal tract (42). Tumors originating from the intestines often present as multilocular cysts or multilocular-solid on ultrasound (43). In our study, all three cases of mucinous cancers were identified as metastatic. Two of these cases had their origins in the appendix, while the third originated from the colon. On US, each lesion appeared as a multilocular cyst and was associated with elevated CA125 levels. There was a risk of misclassifying these lesions as benign if we only depended on US characteristics. However, our adoption of a combined diagnostic strategy was pivotal in accurately recognizing them as malignant. This method played a crucial role in preventing diagnostic oversights and preserving the sensitivity of our diagnostic procedures.

There was a false-negative case that was incorrectly diagnosed as a benign lesion when using the combined diagnosis in the current study. This particular case presented as an unilocular cyst with a solid papillary projection in US. It was histopathologically identified as serous borderline ovarian cancer (SBOT). A study showed that SBOTs typically appear as either unilocular-solid or multilocular-solid cysts (44). Although the positivity rate and average serum level of CA125 generally increase with the progression of stages, a normal serum CA125 level does not rule out the presence of BOT (45). In this instance, the misdiagnosis as benign was influenced by the case being at clinical stage IC with normal CA125 levels. Among the six SBOT cases categorized in O-RADS 4, this was the only case presenting as a unilocular cyst with a solid pp. The other five cases, which displayed multilocular masses with solid lesions, were correctly diagnosed. This indicates that the current diagnostic model has limitations, particularly in the early detection of SBOTs that are ultrasonically present as unilocular with solid characteristics or with <4 pps.

In this study, approximately 14% of AMs were classified as O-RADS 4, with a risk between 10% and 50%. This is similar to the proportion of uncertain masses evaluated by IOTA (46), making it still challenging to determine the malignancy of the masses. We attempted to subclassify the O-RADS 4 lesions, categorizing masses with ultrasound features more indicative of benign nature as 4A, with a malignant risk of 20.9%. The other lesions in O-RADS 4, excluding 4A, were classified as 4B, with a malignant risk of 71.8%. After combining CA125 based on the subclassification, the malignant risks of the two groups were 3.45% and 67.9%, respectively. The combined diagnosis had specificity, PPV, and AUC of 96.5%, 80.2%, and 0.945, respectively. The combined diagnosis improved the diagnostic specificity of O-RADS, which can optimize the intermediate-risk stratification and may be very helpful in deciding surgical strategies and waiting time for surgery.

In a study by Cao (20), O-RADS 4 lesions were further classified into 4A and 4B. Category 4A included multilocular cysts and smooth solid masses, with a malignancy risk rate of 17.02%. Category 4B included unilocular or multilocular cysts with a solid component, and these had a higher malignancy risk rate of 42.57%. Notably, the accuracy significantly increased when the cutoff value was set above 4A. This finding, along with ours, suggested that further stratification and downgrading of O-RADS category 4 lesions can enhance diagnostic accuracy. However, it is important to note that the basis for classification may slightly vary due to the different pathological types of cases included in these studies.

For O-RADS 4 lesions, especially those that are solid or cystic with solid components, MRI is considered for further evaluation of the nature of the lesions. Compared to ultrasound, multiparametric MRI can more accurately characterize the liquid and solid components of AMs, showing good specificity (47). However, MRI needs to be implemented in centers with the necessary software conditions, which remains a challenge for most medical institutions. In addition to requiring a sufficiently long learning curve, radiologists also need to undergo specialized training in O-RADS MRI. Thomassin-Naggara et al. (19) have highlighted several common errors in O-RADS MRI evaluation and analyzed the reasons. In a retrospective study of 1,502 lesions, 139 (approximately 9.2%) were misclassified, mainly due to a misunderstanding of the definition of solid tissue in the lexicon. This error directly reduces the value of MRI in the reassessment of O-RADS 4 lesions. In comparison, our study results show that the combined model improves the diagnostic specificity of O-RADS 4 lesions, providing a new approach for the clinical diagnosis of AMs, which is expected to be further validated in future studies.

The current study has some limitations. Firstly, it was a retrospective study and all analyses were based on static images, which might impact diagnostic accuracy. Secondly, we chose to assess only CA125 in combination with O-RADS due to its common use in clinical settings. Thirdly, the absence of subgroup analyses for premenopausal and postmenopausal patients may impact the generalizability of our results. Fourthly, the limited sample size and the single-center nature of our study may restrict the generalizability of our findings. We will conduct a multicenter prospective study to further validate the use and accuracy of the O-RADS plus CA125 model.

In conclusion, our study demonstrated that the combination of O-RADS and CA125 offers higher diagnostic accuracy and specificity compared to using O-RADS alone. It could be a valuable approach to the risk stratification of AMs for clinical application.

## Data Availability

The raw data supporting the conclusions of this article will be made available by the authors, without undue reservation.
